# Microalgal upgrading of the fermentative biohydrogen produced from *Bacillus coagulans* via non-pretreated plant biomass

**DOI:** 10.1186/s12934-023-02193-0

**Published:** 2023-09-20

**Authors:** Eman S. E. Aldaby, Aya H. A. Mahmoud, Haitham M. El-Bery, Maysa M. Ali, Ahmed A. Shoreit, Asmaa M. M. Mawad

**Affiliations:** 1 Botany and Microbiology Department, Faculty of Science, Assiut University, Assiut, 71515 Egypt; 2 Green Hydrogen Production Laboratory, Chemistry Department, Faculty of Science, Assiut University, Assiut, 71515 Egypt; 3 Department of Biology, College of Science, Taibah University, 42317-8599 Madinah, Saudi Arabia

**Keywords:** *Bacillus*, Biohydrogen, CO_2_ sequestration, Fermentation, Microalgae, Upgrading

## Abstract

**Background:**

Hydrogen is a promising source of alternative energy. Fermentative production is more feasible because of its high hydrogen generation rate, simple operating conditions, and utilization of various organic wastes as substrates. The most significant constraint for biohydrogen production is supplying it at a low cost with fewer impurities.

**Results:**

Leaf biomass of *Calotropis procera* was used as a feedstock for a dark fermentative production of hydrogen by *Bacillus coagulans* AH1 (MN923076). The optimum operation conditions for biohydrogen production were 5.0% substrate concentrationand pH 9.0, at 35 °C. In which the biohydrogen yield was 3.231 mmol H_2_/g dry biomass without any pretreatments of the biomass. A freshwater microalga *Oscillatroia* sp was used for upgrading of the produced biohydrogen. It sequestrated 97 and 99% % of CO_2_ from the gas mixture when it was cultivated in BG11 and BG11-N media, respectively After upgrading process, the residual microalgal cells exhibited 0.21mg/mL of biomass yield,high content of chlorophyll-a (4.8 µg/mL) and carotenoid (11.1 µg/mL). In addition to *Oscillatroia* sp residual biomass showed a lipid yield (7.5–8.7%) on the tested media.

**Conclusion:**

*Bacillus coagulans* AH1 is a promising tool for biohydrogen production avoiding the drawbacks of biomass pretreatment. *Oscillatroia* sp is encouraged as a potent tool for upgrading and purification of biohydrogen. These findings led to the development of a multiproduct biorefinery with zero waste that is more economically sustainable.

**Graphical Abstract:**

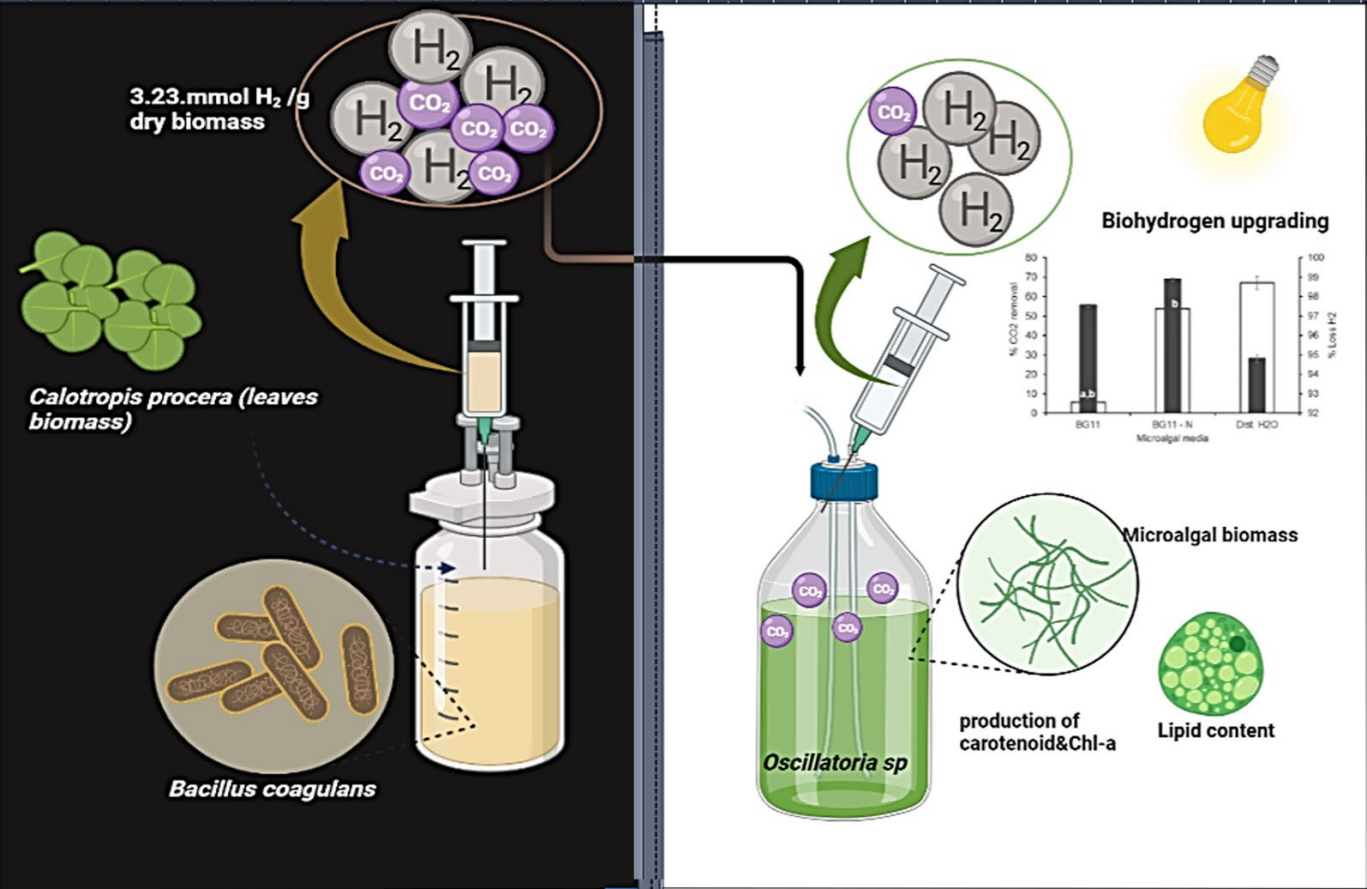

## Introduction

Investigation of new alternative energy sources are promising strategies to address the energy shortage and environmental pollution that threaten social and economic developments [1, 2]. Hydrogen is one of the most valuable renewable carriers, can deliver or store an enormous amount of energy. It can be used in fuel cells to generate electricity, or heat. Hydrogen is characterized as clean, pollution-free and high efficiency and density energy, high, zero-or near zero-emissions operation and reduce greenhouse gases emission [3, 4]. Numerous applications, such as distributed or combined heat and power, backup power, systems for storing and enabling renewable energy, and portable power can be powered by hydrogen and fuel cells. Moreover, hydrogen and fuel cells were used as auxiliary power for transportation, petroleum refining and fertilizer production [4]. As a result, there is much potential for this form of hydrogen production to replace fossil fuels [5]. Biological hydrogen production via fermentative or photosynthetic process is among the diverse technologies for hydrogen production. However, the fermentative production is more feasible as it has a fast rate of hydrogen production, simple operation conditions, and consumption of various organic wastes as substrates [6, 7]. Moreover, fermentative production of hydrogen not only treats the organic wastes but also provides extensive clean energy with low cost [5, 8]. Dark fermentation is the bioconversion of wastes (organic substrates) such as agricultural, and industrial effluents into hydrogen through a series of biochemical reactions catalyzed by bacteria under certain environmental conditions [9]. The major advantage of biohydrogen production through dark fermentation is the consumption of many organic wastes such as cellulosic and lignocellulosic as a substrate and it does not require light. Substrates have a major influence on biohydrogen productivity due to their different biodegradability [10, 11]. Due to their capacity to produce beneficial biopolymers, the latex-bearing plant could be used for many applications [12]. Furthermore, many members of these plants yield active chemicals that are frequently employed in traditional medical practices or toxin production in many countries [13]. *Calotropis procera* (giant milkweed) is a member of the family Asclepiadaceae that has been studied primarily because of its variable medicinal properties [14]. It has evergreen leaves, extensively disseminated in the world [15, 16]. Other research have revealed that *C. procera* contains significant levels of hydrocarbons. [16–18]. *Calotropis procera* was a potential plant for bioenergy and biofuel production in semiarid regions of the world [19, 20] as it is a fast-growing plant with easily accessible biomass that is not normally fed by cattle due to latex toxicity [15].

The limitation of biohydrogen production via dark fermentation is the release of minor impurities such as CO_2,_ H_2_S, N_2_ and water vapour that reduce the quality of produced hydrogen and constrain its industrial applications. Carbon dioxide which included in biogas reduces the burning velocity which consequently impacts the performance of the engine as well as decreases the peak pressure and the maximum power inside the cylinder [3, 21]. Biogas containing more than 45% carbon dioxide causes harsh and irregular running of the engine. Therefore, reduction of carbon dioxide content will certainly improve the quality of biogas [22, 23]. Microalgae and cyanobacteria are believed to solve the problem of biohydrogen gas impurities due to their potential for CO_2_ sequestration [24]. They remove CO_2_ from the atmosphere with greater efficacy than conventional energy crops. The CO_2_ efficacy may reach 99%, which corresponds to 1.8 kg of CO_2_ per kilogram of dried biomass [25, 26]. In addition, they are anticipated to serve as the raw material for upcoming multiproduct biorefineries. Owing to their rapid growth, substantial metabolite composition, stress-tolerance mechanism, efficacy in remediation of wastewater, highest CO_2_ fixation rate, and production of valuable metabolites with industrial, environmental, and pharmaceutical significance [27–30] The majority of microalgal biomass (> 75%) is composed of lipids and carbohydrates. Carbohydrates derived from microalgae contribute equally to the biofuel industry through fermentation or acid treatment to produce bioethanol. Oleaginous microalgae can store 20–50% of their desiccated cell mass in oil, which is 300 times more than conventional energy crops [31, 32]. Several microalgal species can be stimulated to produce lipids by depriving themselves of nutrients [33]. After lipid extraction, the desiccated biomass can be utilized for bioethanol production, biochar production, and biofertilizer carriers [34]. Moreover, the pigments produced from microalgae can be used for industrial purposes [35]. Therefore, the main objectives of this study are (i) microbial screening and isolation of biohydrogen producing bacteria via non-pretreated biomass of *Calotropis procera*, (ii) biohydrogen production using *Bacillus coagulans* AH1 including optimization of fermentation conditions, (iii) upgrade of biohydrogen using microalgal fermentation, iv) assessment of microalgal usability for lipids, pigments.

## Materials and methods

### Samples collection

Leaves biomass of *C. procera* were collected from Wadi- Al-Assiuty-Assiut governorate, Egypt and prepared as described by [19]. The granular sludge sample was collected from the wastewater treatment plant in Arab Al-Madabegh region, Assiut-Egypt. The samples were kept in plastic sacs and used as a source of bacterial inoculum.

### Enrichment, isolation and purification of hydrogen-producing bacteria

The fermentative biohydrogen-producing bacteria were isolated from the collected sludge sample. About 10% w/v of sludge was seeded in a sterilized anaerobic medium that was prepared according to the method described by [36]. The medium was supplemented with 10% of dried leaves biomass of *C. procera* as a substrate for biohydrogen production at pH 7.0 under strictly anaerobic conditions. It was incubated at 35 °C for 10 days under aseptic dark conditions with continuous shaking at 120 rpm to provide better contact with the substrate. The produced gas was received and collected in the graduated sterilized syringe. The previous step was repeated three times by placing 10% of the growing bacteria in a new anaerobic media to perform enrichment under the same previously mentioned conditions.

After the third enrichment step, a volume of 5 mL of the fermented mixture was re-seeded into a 50 mL serum bottle consisting of broth reinforced clostridial medium (RCM) [37, 38] for isolation of the potential hydrogen-producing isolates under anaerobic conditions. The composition of RCM per 1000 mL was: 3.0 g yeast extract, 10.09 g beef extract, 10.0 g peptone, 3.0 g sodium acetate, 5.0 g sodium chloride, 5.0 g glucose, 1.0 g soluble starch and 0.5 g cysteine-hydrochloride, pH was adjusted to 6.8. The bottles were incubated at 35 °C with continuous shaking at 120 rpm for 24 h. After that 100 µl was streaked onto RCM agar covered with sterilized paraffin oil and incubated at 35 °C for 24 h. The pure bacterial isolates were obtained by repeated streaking on RCM agar covered with sterilized paraffin oil. The purified isolates were separately preserved for further use.

### Molecular genetic characterization of the bacterial isolates

The bacterial isolates were genetically characterized based on 16S rRNA gene sequencing after extraction of total genomic DNA [39, 40]. Extraction of the genomic DNA of the isolates was carried out at the Molecular Biology Research Unit, Assiut University. Using universal primers designed to amplify a 1500 bp segment of the 16S rRNA gene, the conserved area of the gene was amplified by polymerase chain reaction (PCR) (Solgent Co., Ltd, Bio-Industry Development Site, 63–10 Hwaan-Dong, Yuseong-Gu, Daejeon, South Korea). The reverse primer was 1492R(AGAGTTTGATCCTGGCTCAG), while the forward primer was 27F (CGGCTACCTTGTTACGACTT). The obtained sequences of the isolates were aligned and compared with the known 16SrRNA gene sequences in the GenBank database using the BLAST search at www.ncbi.nlm.nih.gov/blast/Blast.cgi.

A phylogenetic tree was developed to determine the isolate's taxonomic classification using MEGA 4.0's neighbour-joining approach. Phylogenetic trees were derived from 16S rRNA gene sequences which built in the context of 16S rRNA gene sequences from different bacterial strains deposited from GenBank [41, 42]**.**

### Optimization of fermentative biohydrogen production

The bacterial cells were previously cultivated in RCM at 35 °C, pH 7.0 and shaking at 120 rpm, under dark strictly anaerobic conditions (by purging with argon). A volume of 20 mL of bacterial suspension was anaerobically placed in a sterilized vail, centrifuged at 5000 rpm for 10 min, and resuspended in phosphate buffer saline (PBS). The batch experiments were performed in 120 mL sterilized serum bottles with a working volume of 50 mL of sterilized anaerobic medium supplemented with 0.5 g dried leaves powder of *C. procera* after autoclaving and inoculated with 10% v/v pre-growing bacterial cells (OD660 = 1.3). The bottles were sealed well with sterilized air-tight rubber caps and parafilm, and incubated in the dark at 35 °C. Then purged with argon gas to drop the dissolved oxygen concentration down to zero. During the incubation time (10 days), the produced gas was received in a sterilized graduated syringe.

The concentration of produced biohydrogen was determined by gas chromatography (GC) (Shimadzu, GC- 2014) equipped with a thermal conductivity detector (TCD) and Shin Carbon packed column (ST 80/100 2 m, 2 mm ID). Argon was used as carrier gas.

To assess the optimum nutritional and environmental operation conditions for maximum biohydrogen productivity, a conventional one-factor-at-a-time (OFAT) method was applied. The batch experiments were performed as previously mentioned at different substrate concentrations (2.5, 5.0, 7.5, 10.0, 12.5 and 15.0%) with pH 7 at 35 °C. For determination the optimum pH, the medium pH was adjusted to 3.0, 5.0, 7.0, 9.0 or 11.0 before autoclaving using dilute HCl or NaOH 10% of substrate was added at 35 ℃. The fermentative media with a substrate concentration of 10% and pH 7.0 was incubated at (25, 30, 35, 40 and 45 °C) to determine the impact of environmental factors on biohydrogen production. The bottles were incubated for 10 days with continuous shaking at 120 rpm. A control sample was performed using a fermentative medium containing either bacterial inoculum (without substrate supplementation) or *C. procera* biomass (without bacterial supplementation). An additional blank assay with only an anaerobic medium without substrate or inoculum was also conducted.

After the assesment of the optimum value of each factor, the dark fermentation process was performed to evaluate the amount of producing hydrogen at the optimum conditions.

### Biological upgrading of the produced biohydrogen by microalgae

A filamentous microalgal isolate *Oscillatoria* sp. was previously isolated from the Nile River canal, in Assiut, Egypt. It was deposited in the World Data Center for Microorganisms at Suez Canal University Fungarium collection WDCM-1180 with strain ID: *Oscillatoria* SCUF0000351. *Oscillatoria* sp. was enriched in BG11 medium consists per g/L 1.5; NaNO_3_, 0.04; K_2_HPO_4_, 0.075; MgSO_4_·7H_2_O, 0.036; CaCl_2_·2H_2_O, 0.006; Citric Acid·H_2_O, 0.006; ferric ammonium citrate, 0.001; Na_2_EDTA·2H_2_O, 0.02; Na_2_CO_3_ and 1 mL of trace element mixture at pH 7.4 [43]. The microalgal cells were incubated at 35 °C under a white, fluorescent lamp (2000–3000 lx intensity) as a light source.

**Fig.1:**
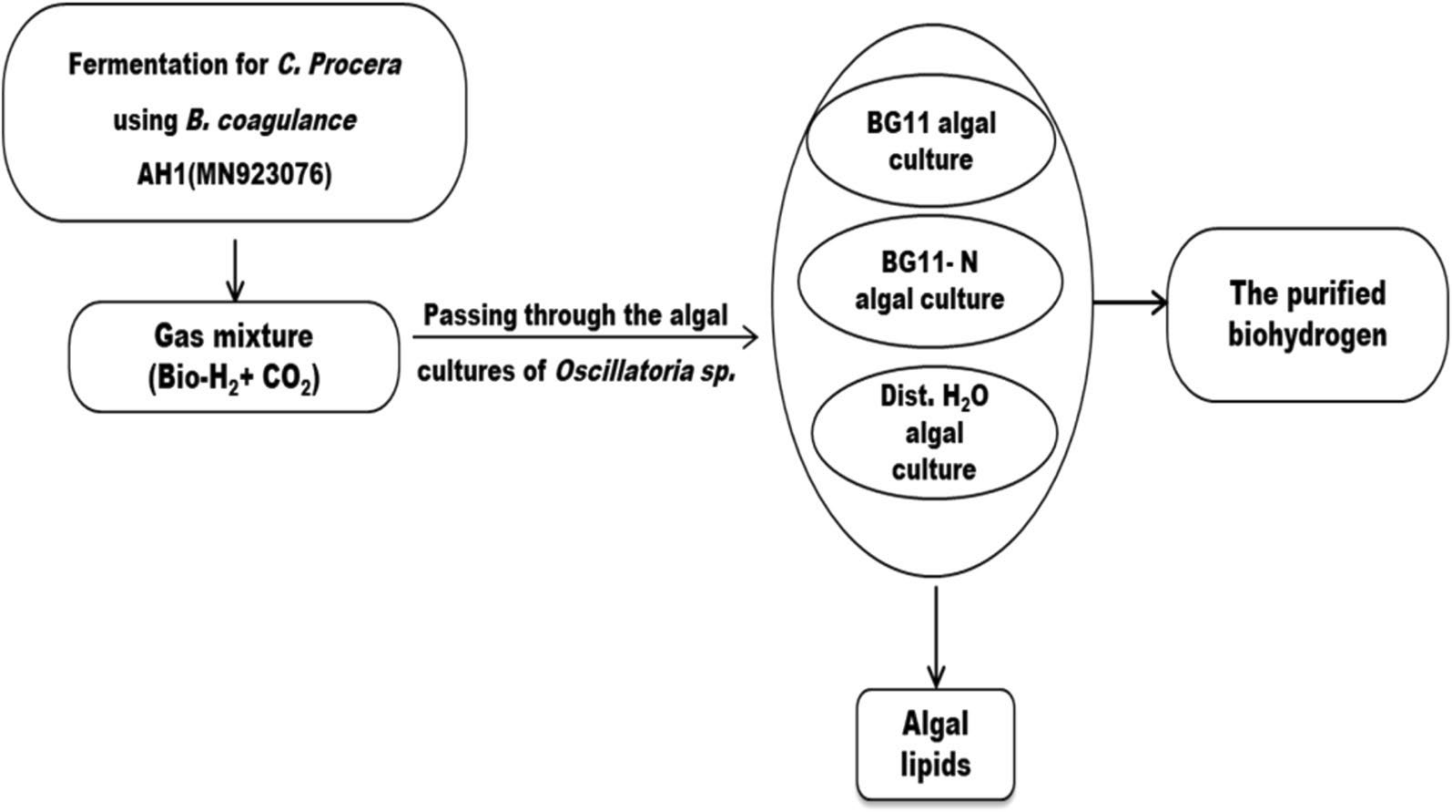
Flowchart for biohydrogen purification by *Oscillatoria* sp

For determine the gas upgrading potential, the microalgal cells were cultivated in air-tight rubber cap stoppered glass bottles containing three different media: BG11, nitrogen-deprived BG11 medium (BG11- N) or distilled water. The gas mixture (biohydrogen + CO_2_) that was previously produced from AH1 bacteria at the optimum conditions was then passed over to the cultivated cyanobacterial cells. They were incubated under a white, fluorescent lamp (2000–3000 lx intensity) at 35 °C and 150 rpm in an orbital shaker as illustrated in the flowchart Fig. 1. The impact of the passed gas mixture on the growth indices and the physiological activities of the microalgal cell was investigated.

### Determination of the removal efficiency of carbon dioxide

The concentration of gas mixture (H_2_ and CO_2_) before (inlet) and after (outlet) passing through *Oscillatoria* sp. cultures was determined by gas chromatography (GC). The inlet and outlet gases were expressed as mmol (H_2_ or CO_2_)/g of microalgal dry weight (DW). The biohydrogen to carbon dioxide ratio (H_2_/CO_2_) was estimated before and after the purification process as well as the estimation of the capability of CO_2_.

The removal efficiency of *Oscillatoria* sp was expressed by the consumed percentage of the inlet CO_2_ [44] as the following equation:

1 $${\mathbf{CO}}_{{\mathbf{2}}} {\mathbf{removal}} \, {\mathbf{efficiency}} = \, [({\text{Inlet CO}}_{{2}} {-}{\text{ Outlet CO}}_{{2}} ) \, /{\text{ Inlet CO}}_{{2}} ] \, \%$$

### Determination of physiological and growth indices of *Oscillatoria* sp.

#### Determination of growth kinetics of *Oscillatoria *sp.

A volume of 50 mL of the cyanobacterial cells were harvested by centrifugation for 15 min. at 5000 rpm. The dry weight of cyanobacterial cells was determined after an overnight dry at 60 °C and it was expressed as mg/mL cell suspension. At the termination of each batch culture, the growth kinetics were carried out on a dry mass basis using the gravimetric technique previously used [45]. The biomass productivity (P biomass (mg mL^−1^d^−1^) was estimated by applying the following equation:

2 $${\text{P}}_{{{\text{biomass}}}} = \, \left( {{\text{W}}_{{\text{y}}} - {\text{W}}_{{\text{x}}} } \right)/{\text{reaction volume }}\left( {{\text{mL}}} \right)$$

where W_x_ and W_y_ were the initial and the final biomass concentrations at the end of the incubation period.

The specific growth rate (μ) was measured in terms of day^−1^ via the following equation:

3 $$\mu \, = {\text{ ln }}\left( {{\text{W}}_{{\text{y}}} /{\text{W}}_{{\text{x}}} } \right)/\left( {{\text{t}}_{{\text{y}}} - {\text{ t}}_{{\text{x}}} } \right)$$

where t_y_ and t_x_ were the time of harvesting and start time of cultivation, respectively. The doubling time (T_d_) for microalgae could be derived from Eq. (3) as follows:

4 $${\text{T}}_{{\text{d}}} = \, \mu \, \left( {{\text{t}}_{{\text{y}}} - {\text{ t}}_{{\text{x}}} } \right)/{\text{log2}}\left( {{\text{W}}_{{\text{y}}} /{\text{W}}_{{\text{x}}} } \right)$$

#### The potential captivity of carbon dioxide by *Oscillatoria* sp.

In terms of CO_2_ consumption by the microalgal biomass, the ability of microalgae to fix CO_2_ for conducting photosynthesis was assessed regularly. According to an equation developed from the average molecular formula of algal biomass CO0.48, H1.83, N0.11, P0.01 [46], the CO_2_ fixation rate (mg/mL d) was calculated, as follows:

5 $${\text{PCO}}_{{2}} = { 1}{\text{.883}}\, \times \,{\text{P}}_{{{\text{biomass}}}}$$

#### Estimation of photosynthetic pigments content in *Oscillatoria* sp.

Chlorophyll was investigated to determine the photosynthetic efficiency while carotenoid pigment was investigated to detect the potential of biotechnological applications. They were extracted by absolute methanol and quantified according to [47]. A volume of 2 mL of the cell suspension was centrifuged at 5000 rpm for 10 min to obtain the cell pellet. Then pigments were resuspended in absolute methanol for 10 min for pigment extraction. The sample was then kept for 24 h at 4 °C in the dark. It was centrifuged for 10 min at 5000 rpm to remove the cell debris. The supernatant was determined at 665 nm and 470 nm against blank (methanol). The chl-a and total carotenoid contents were estimated based on the following:

6 $${\text{Chl}} - {\text{a }}\left( {\mu {\text{g}}/{\text{mL}}} \right) \, = { 13}.{43 }\left( {{\text{A665}}} \right){\text{ v}}/{\text{b V}}$$

7 $${\text{Carotenoids }}\left( {\mu {\text{g}}/{\text{mL}}} \right) \, = { 4}.{4 }\left( {{\text{A47}}0} \right){ 1}0)/{\text{b V}}$$

where A665 and A470 are the absorbance values at 665 nm and 470 nm, respectively, against a blank; v was the volume of applied solvent (mL), b was the spectrophotometric cell length (1 cm), and V is the sample volume (mL).

#### Determination of the potential biodiesel production from *Oscillatoria* sp.

After the upgrading process, the potential lipid content produced form *Oscillatoria* sp. was determined according to [48]. A weight of 50 mg of *Oscillatoria* sp. biomass was harvested and homogenized in 4 mL ice—chilled chloroform–methanol (2:1) to release lipids. After that it was separated into chloroform and aqueous methanol layers by the addition of 4 mL ice–chilled 1M MgCl_2_ solution. The lower chloroform layer which contained most of the algal lipids was drawn off from the tube via a long syringe. Lipids were transferred into dry test tube and then evaporated and weighted to obtain the lipid yield and content. The lipid yield calculated as % (w/w) culture.,

8 $${\text{Lipid content }}\left( \% \right) \, = \, \left[ {\left( {{\text{mg lipid }}/{\text{ mg dry mass}}} \right) \, *{1}00} \right]$$

### Statistical analysis

All experiments were performed in triplicate sets. Statistical analysis of data was performed using a one-way ANOVA test (Analysis of variance) by SPSS program version 25. Dunken value was determined at 0.05 level.

## Results

### Isolation, molecular identification, and phylogenetic analysis of the H_2_-producing isolate

**Fig. 2:**
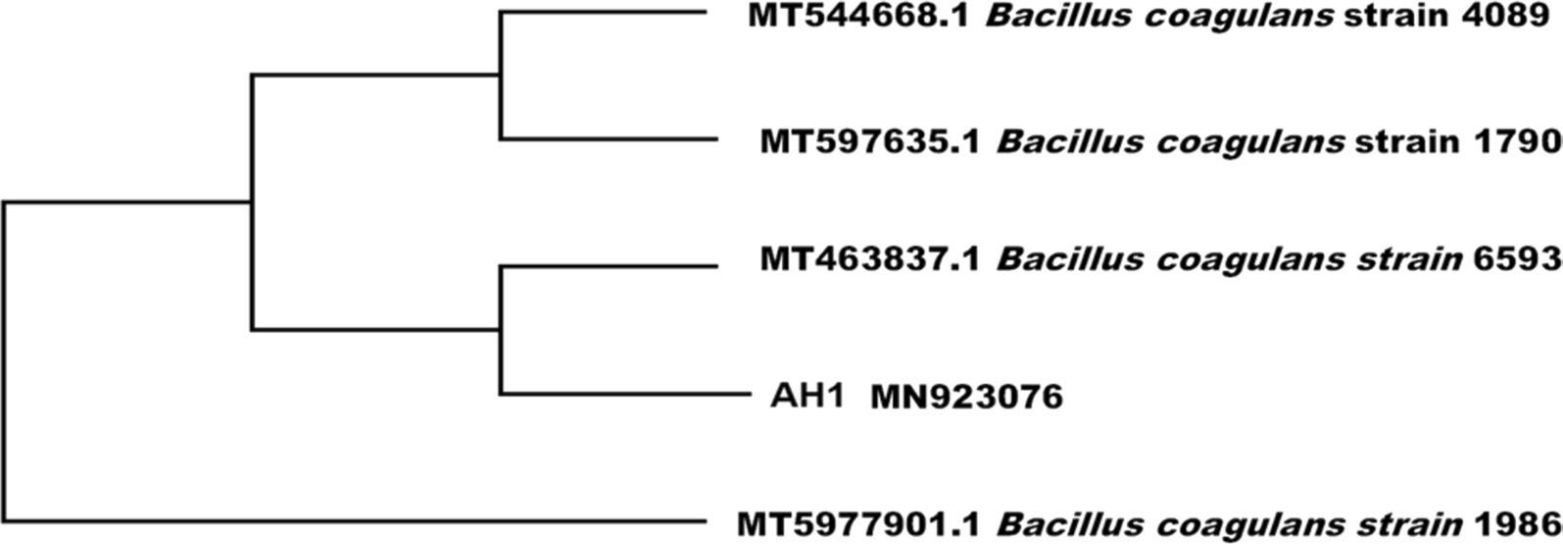
Phylogenetic relationship between the strains AH1 and other 16S rRNA gene sequences of published *Bacillus coagulans* strains

Ten anaerobic bacteria isolates were isolated from the sludge sample. The strain AH1 exhibited a high biogas productivity. Therefore, it was selected for further studies. The nucleotide sequence of the hydrogen-producing bacterial isolate has been deposited in the GenBank nucleotide sequence database under the name *Bacillus coagulans* strain with the accession Number MN923076. As analyzed by BLAST, the sequence of the 16S rRNA gene of the isolated strain AH1 showed high identity (99.93%) with *Bacillus coagulans* MT463837 in the GenBank as illustrated in Fig. 2.

Several sequences relevant to various *Bacillus* species were chosen from the Genbank database for the building of the phylogenetic trees to corroborate the location of the strain AH1 in the phylogeny. The tree revealed that *B. coagulans* and strain AH1 belonged to the same clade cluster Fig. 2. As a result, *Bacillus coagulans* was determined to be strain AH1. The strain AH1 was deposited in at Suez Canal University Fungarium culture collection WDCM-1180 under strain ID: *Bacillus* SCUF0000352.

### Determination of the operation conditions for biohydrogen production

#### Impact of substrate concentration on Biohydrogen productivity

**Fig. 3:**
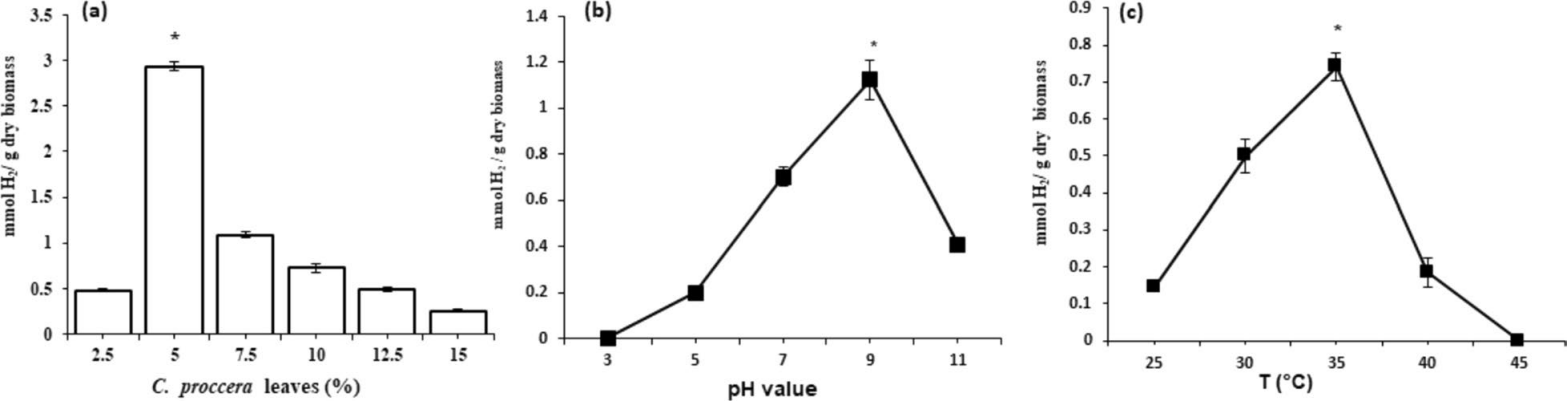
Biohydrogen production from *Bacillus coagulans* using *Calotropis procera* different substrate concentrations **a**, different pH **b** and different temperatures **c**. *; represents a significantly different at (p ≤ 0.05) based on Duncan’s multiple range test

The concentration of the produced biohydrogen was investigated at different concentrations of *C. procera* leaves powder (2.5, 5.0, 7.5, 10.0, 12.5, and 15.0%). The amount of producing hydrogen from *B. coagulans* AH1 was 0.48, 2.94, 1.09, 0.73, 0.49, and 0.26 mmol H_2_/ g dry biomass, respectively. It was observed that the optimum production of biohydrogen was significantly (p ≤ 0.05) determined at 5.0% of substrate concentration and above this concentration the biohydrogen productivity dramatically reduced as illustrated in Fig. 3a**.**

#### Impact of initial pH on biohydrogen productivity

Various pH values 3.0, 5.0, 7.0, 9.0 and 11.0 were assessed to investigate the optimum value for biohydrogen production at 35 °C, and 5% of substrate concentration. The concentrations of the produced biohydrogen were 0.0, 0.2, 0.7, 1.12, and 0.41 mmol H_2_/g dry biomass at the assessed pH values, respectively. It was noticed that the acidic pH of 3.0 significantly (p ≤ 0.05) inhibited the biohydrogen productivity however, the optimum productivity was recorded at an alkaline pH value; of 9.0 as shown in Fig. 3b.

#### Impact of temperature on biohydrogen productivity

The biohydrogen production was investigated at different temperatures (25, 30, 35, 40, and 45 °C) at pH 7.0 and 10% of the substrate concentration the concentration of produced biohydrogen by AH1 was 0.14, 0.50, 0.74, 0.18, and 0.0 mmol H_2_/g dry biomass at the tested temperatures, respectively. It was noticed that the highest (p ≤ 0.05) biohydrogen productivity was estimated at 35 °C while the productivity was completely inhibited at 45 °C as illustrated in Fig. 3c**.** On the other hand, the low temperature 25 °C significantly (p ≤ 0.05) showed reduced productivity compared to the high one Fig. 3c.

#### Biohydrogen productivity under optimal operation conditions

Based upon the previous optimization results, the optimum conditions that achieved highest biohydrogen productivity were 5% of substrate, pH 5.0 and 35 ℃. The concentration of the biohydrogen under the optimum conditions was 3.231 mmol H_2_/g dry biomass.

### Biological upgrading of biogas using *Oscillatoria* sp.

#### Biohydrogen to carbon dioxide ratio before and after the upgrading process

**Fig. 4:**
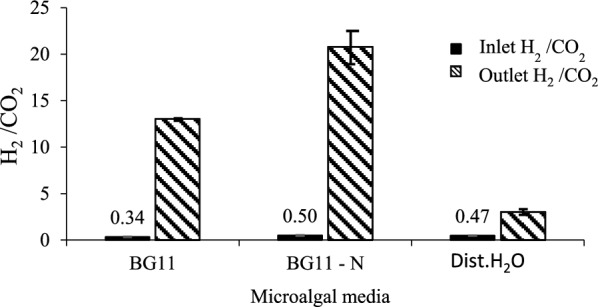
Biohydrogen to carbon dioxide ratio before and after purification using different algal cultures media

The results in Fig. 4 displayed that the amount of inlet biohydrogen to the inlet carbon dioxide ratio (H_2_/CO_2_) was 0.34, 0.50 and 0.47 while the amount of outlet H_2_/CO_2_ ratio was 13, 21 and 3, for the microalgal culture BG11^+g^, BG11-N^+g^ and dist. H_2_O^+g^, respectively. This result showed that there was a significant difference (p ≤ 0.05) between the H_2_/CO_2_ ratio before and after passing gases on microalgal cultures, where the outlet ratio exhibited a significant (p ≤ 0.05) higher than the inlet one. It was also noticed that the highest H_2_/ CO_2_ ratio was detected in BG11-N medium, while the lowest H_2_/ CO_2_ ratio was detected in dist. H_2_O microalgal medium despite it was still higher than the inlet ratio.

#### Determination of certain concentrations of the inlet and outlet gases

**Fig. 5:**
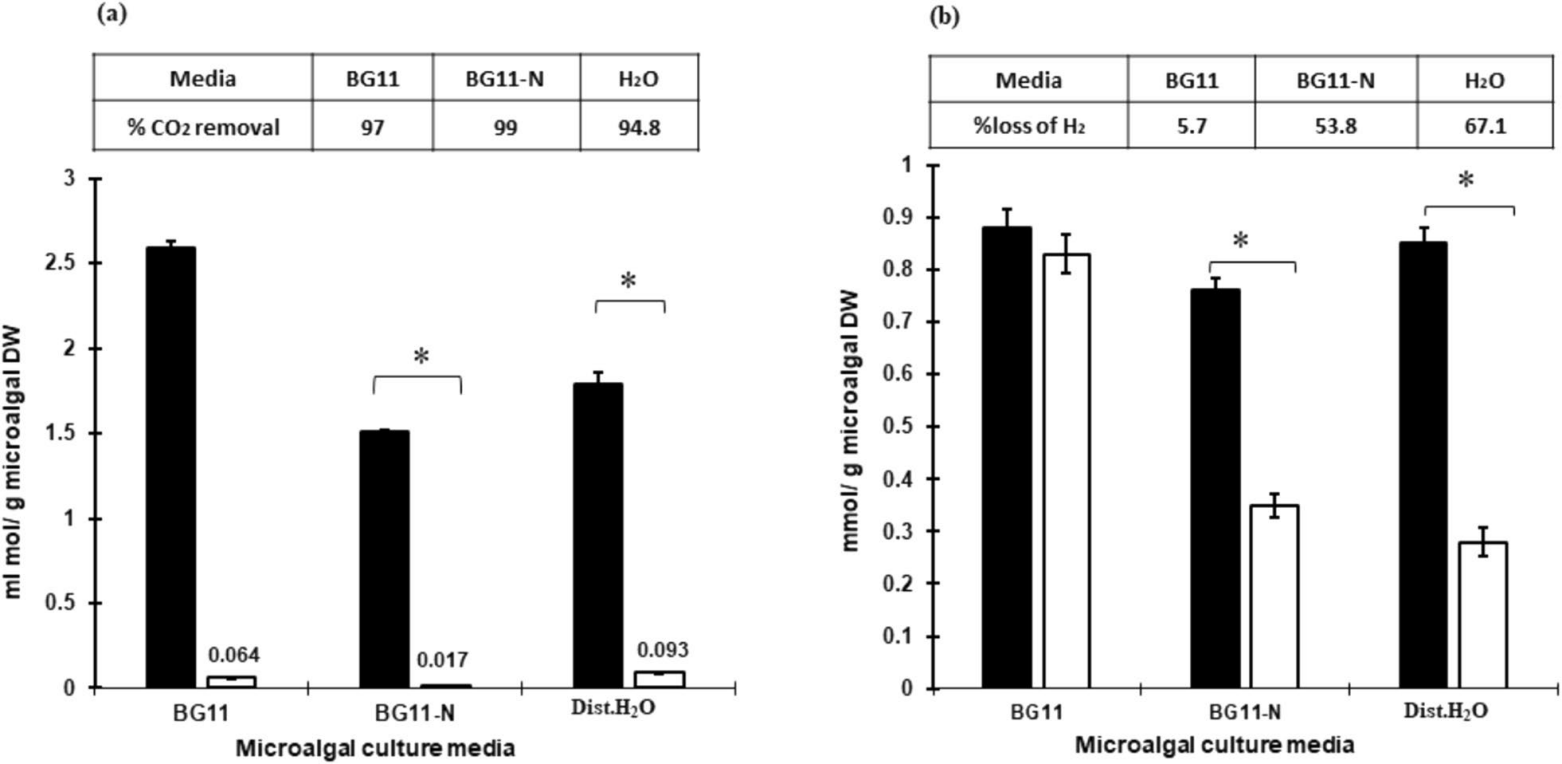
The concentration of inlet (black column) and outlet (white column) CO_2_ (**a**) and H_2_ (**b**) through upgrading process by *Oscillatoria* sp. that grown on the microalgal cultures BG11, BG11– N and dist. H_2_O. *; represents a significantly different at (p ≤ 0.05) based on Duncanꞌs multiple range test. Tables represent the performance of biohydrogen upgrading process; % removal of CO_2_ (**a**) and % loss of H_2_(**b**)

Carbon dioxide bio-fixation using microalgal-based technology is an economically feasible method for upgrading the biohydrogen. The capability of *Oscillatoria* sp. to purify and upgrade the biohydrogen produced by anaerobic bacterial fermentation via capturing of CO_2_ was investigated. The results in Fig. 5a&b illustrated (inlet) gases through each microalgal media and the outlet gases after the microalgal upgrading process. The inlet CO_2_ was 2.59, 1.51, 1.79 mmol CO_2_/g microalgal DW, while after passing the gases through the *Oscillatoria* sp., the outlet CO_2_ was 0.064, 0.017, 0.093 mmol CO_2_/g microalgal DW for BG11^+g^, BG11-N^+g^, and dist. H_2_O^+g^ culture media, respectively. It was noticed that there was a significant (p ≤ 0.01) reduction in the amount of outlet CO_2_ compared with the outlet CO_2_ Fig. 5a_._

On the other hand, the amount of inlet and outlet biohydrogen Fig. 5b. The inlet biohydrogen was 0.88, 0.76, 0.85 mmol H_2_/g microalgal DW while the outlet biohydrogen was 0.83, 0.35, 0.28 mmol H_2_/g microalgal DW for the microalgal media BG11^+g^, BG11-N^+g^ and dist. H_2_O^+g^ culture media, respectively. It was observed that the BG11 medium exhibited a conserved medium that did not negatively impact the amount of biohydrogen.

### Performance of biohydrogen upgrading process

The results in Fig. 5 illustrated the performance of the upgrading process that was estimated based upon the inlet and outlet gases results. The cultivation of microalgae in BG11-N medium significantly (p ≤ 0.05) enhanced the removal of CO_2_ by 99% compared with H_2_O-grown microalgal isolate 94.8%. It was observed that there was a missing percentage of biohydrogen during the upgrading process in different microalgal culture media. The microalgal dist.H_2_O culture medium exhibited a significant (P ≤ 0.05) increase in the biohydrogen loss (67.1%), while the microalgae cultivated in BG11 medium exhibited a non-significant loss (5.7%) in the amount of biohydrogen compared to the inlet percentage. However, it exhibited a significant decrease in the loss of biohydrogen when compared with BG11-N and H_2_O microalgal media Fig. 5a, b.

### Impact of upgrading process on the microalgal physiological activities

The results in Table.1 showed that the maximum biomass production of 0.21 mg/mL, 0.13 and 0.18 g L- 1 (dry mass basis) were observed when the microalgae were cultured in BG11^+g^, BG11-N^+g^ and H_2_O^+g^, respectively, which indicated that *Oscillatoria sp.* performed better growth in BG11, which is often noticed in cyanobacterial isolates. On the other hand, the bottles that were not exposed to the gas mixture showed a negative impact on the growth and biomass production of microalgae in three culture media.

**Table1:** The growth parameters, carbon dioxide fixation rate and pigmentation of microalgal gown on different culture media

Algal culture	Growth parameters			PCO_2_ (mg/mL/d)	Pigmentation (µg/mL)	
	Biomass production (mg/mL)	Specific growth rate(d^−1^**)**	Td (d)		Chl-a	Carotenoid
BG11	−0.4	−0.01	−0.03	ND	2.8	4.7
BG11^**+g**^	0.21^*^	0.023	0.076	0.43^*^	4.8^*,**^	11.1^*^
BG11-N	−0.1	−0.008	−0.027	ND	1.03	2.34
BG11-N^**+g**^	0.13	0.013	0.044	0.24	2.43	4.7
H_**2**_**O**	−0.5	−0.06	−0.2	ND	0.93	1.44
H_**2**_**O**^**+g**^	0.08	0.005	0.02	0.14	1.13	2.6

ND: not detected, ^+g^: gas mixture

^*^,**: Values with different superscript asterisks indicate a significant difference at

p < 0.05 according to the Duncan’s multiple range tests

The maximum specific growth rate (0.023/d) and doubling time (0.076 d) were observed in microalgae growing in BG11^+g^ Table 1. The rate of carbon dioxide fixation was detected in three tested culture media, the results showed that the BG11^+g^ exhibited significant (p ≤ 0.05) maximum CO_2_ fixation rate (0.42 mg/mL/d) when compared with BG11-N^+g^ or H_2_O^+g^.

Microalgal photosynthetic pigments play a role in industrial applications. The results in Table 1 showed that the cultivation of *Oscillatoria* sp. in BG11^+g^ enhanced the production of both chlorophyll-a and carotenoid pigments to be 4.8 and 11.1 µg/mL when the content of the initial pigment before passing of gas mixture was 1.1 and 2.5 µg/mL, respectively. These results were significantly (p ≤ 0.05) high when compared with other tested microalgal culture media. The microalgae that were cultured in H_2_O^+g^ showed no significant increase in the chlorophyll-a and carotenoid content (1.13 and 2.5 µg/mL) when compared with the initial ones 1.1 and 2.5 µg/mL, respectively.After the end of the purification process, the microalgal cells grown in different culture media used for biohydrogen upgrading could be reused for another process or extraction of lipids for prospective biodiesel production. The results in Table 2 showed that the lipid content was 8.7, 7.5 and 7.4% for the microalgal cultures BG11^+g^, BG11-N^+g^ and dist.H_2_O^+g^ respectively. Lipid yield was 2.9, 2.6 and 2.3 µg/mL respectively.

**Table 2:** Lipid yield and Lipid content which extracted from *Oscillatoria* sp*.* from the used cultures media BG11^+g^, BG11—N^+g^ and dist. H_2_O^+g^

Algal condition	Lipid yield (µg/ml)	Lipid content (%)
BG11^+g^	2.9	8.7
BG11—N^+g^	2.6	7.5
Dist. H_2_O^+g^	2.3	7.4

(^+g^) Algal culture media was used for purification

## Discussion

The fermentation method of producing biohydrogen is proven to be efficient since it is safe for the environment, reduces the need for fossil fuel consumption, and minimizes pollutants. However, biohydrogen production via dark fermentation is complex and influenced by many factors including type and pretreatment of substrate, type and source of inoculum, pH and temperature [2]. Regarding the inoculum type, *B. coagulans* AH1 (MN923076) is a gram-positive, facultative anaerobic bacteria isolated from wastewater sludge. As mentioned by [50], the facultative anaerobic fermentative microorganisms were cost effective during the biohydrogen production compared with strictly anaerobes. As they can enhance the anaerobic condition of the medium by fast utilization of the dissolved oxygen. In addition, many literatures discussed the effective role of sludge inhabiting bacteria for the fermentative production of hydrogen [2, 51]. In addition, the deceasing in the biohydrogen productivity when the biomass exceeded 5% may be attributed to the enhancement of metabolic and enzymatic activities of the fermentative bacteria under this concentration (5%) [4, 7, 52]. However, at high substrate concentrations, it might be rapidly converted to hydrogen and /or volatile organic acids that cause the drop in pH, consequently decreasing the metabolic activity of fermentative bacteria and reducing the produced H_2_. Moreover, it caused high hydrogen partial pressure in the fermentation medium that would restrict the hydrogen production efficiency [53]. In addition, an excessive amount of the substrate increased osmotic pressure and thus inhibited the growth of hydrogen-producing microbes [4, 54]. On the other hand, at low substrate concentrations, a large amount of it may be consumed for microbial growth rather than hydrogen production [4]. Notably, in this study, the biomass of *C. procera* was directly supplemented as a substrate for bacterial dark fermentation for H_2_ production without any prior treatment. Based upon our previous study, the leaf biomass of *C. procera* was used as a main substrate for the production of bioethanol after acid-alkaline pretreatment as well and it was reported to be applied as a promising feedstock for biodiesel production [19]. However, in the current study, the direct application of plant biomass led to avoiding the negatives of the different pretreatment strategies (i.e. high cost, energy and time consumption) [49].

The most crucial variables in the anaerobic fermentation process are temperature and pH. These variables affect the synthesis of biohydrogen and microorganisms that produce H_2_ [55]. In the current study, the amount of biohydrogen reduced at low pH may be due to the inhibition of hydrogenase activity, which is a key enzyme in the biohydrogen production process or inhibition of the metabolic activity of bacteria [55–58]. Moreover, [59] reported that the low pH value stimulated the solventogenesis and methanogenesis that suppress the production of hydrogen during fermentation. The optimum pH 9.0 of AH1 strains for maximum production of hydrogen was compatible with that recorded by [60, 61] using glucose as the sole substrate, but it was higher than the value of H_2_ at pH 7.5 that demonstrated by [55] using molasses. According to [62], pH levels above 10.0 had a toxicity impact on the microorganisms, which explained the decrease of biohydrogen production at pH 11.0 in the current study.

Besides that, dark fermentative biohydrogen production could be conducted at a broad range of temperatures, 25–80 °C [63]. The ambient temperature average for hydrogen production is from 30 to 49 °C and it is also preferred in terms of expenditures and other technical features [22]**.** Biohydrogen production under mesophilic conditions is cost effective, less energy consumption and easy to regulate on a large scale [52]. The production under mesophilic conditions (30–35 °C) has been reported by [60, 64] which was agreed with the current results. A lower culture or higher culture temperature deactivated or denaturized the microbial enzyme system and consequently decreased the biohydrogen production [65, 66]. The optimum conditions for biohydrogen production from *Bacillus coagulans* IIT-BT S1 was reported by (Kotay, and Das, 2007) were pH 6.5, temperature 37 °C and initial glucose concentration of 2% (w/v), to produce 2.28 molH_2_/mol glucose. The data in Table 3 showed the optimal conditions of *B. coagulans*, for biohydrogen production from *C. procera* leaves compared to those reported in other studies.

**Table 3:** Biohydrogen production from *C. procera* leaves under the optimal conditions of the bacterial strain AH1 compared to the other bacterial cultures that reported in other studies

Culture	Substrate	T °C	pH	H_2_ yield	Refs
*B. coagulans* strain AH1	Leaves of *C. procera*	35	9.0	3.231 mmol H_2_ /g dry biomass	This study
*Escherichia coli* WDHL	Wheat straw	28–46	5.5–7.5	0–269.2 mL H_2_/g TRS	[75]
Mixed culture (Anaerobic sludge from an UASB reactor located at a confectionery)	Oat straw	35	7.0	1.10–2.39 mol H_2_/mol reducing sugars	[76]
*Clostridium beijerinckii* KCTC 1785	Sorghum rusk	30–45	6.0–7.5	1.05 mol H_2_/mol reducing sugar	[77]
*Clostridium butyricum TM-9A*	Molasses	37	7.5	46 mmol H_2_/L	[55]
*Enterobacter cloacae IIT-BT08*	Glucose	36	6.0	2.2 mol H_2_/mol glucose	[78]
*Clostridium roseum* ATCC 17,797	Cashew apple bagasse	38	5.5	0.08–1.89 mL H_2_/g biomass	[79]
*Enterobacter aerogenes* NBRC 13534	Coconut husk	37	7.0	0.104–0.175 mol H_2_/ mol reducing sugar	[80]
Mixed culture (Anaerobic sludge from a wastewater treatment plant of a baker yeast company)	Wheat powder	37	6.0	0.6–1.6 mol H_2_/mol total sugar	[81]
Anaerobic sludge	Glucose 14.01 g/L	33	5.61	2.54 mol H_2_/mol glucose	[82]

Carbon dioxide including biohydrogen represents the major obstacle to application of biogas as an alternative fuel [2, 3]. In this study a blue-green microalgae *Oscillatoria* sp was used for the sequestration of carbon dioxide from the biohydrogen gas produced from bacterial dark fermentation. The produced gas mixture (H_2_ + CO_2_) passed through three different culture media containing microalgal cells. The increase in hydrogen to carbon dioxide ratio after passing of the gas mixture (outlet H_2_/CO_2_) through the microalgal media was due to the reduction in the amount of carbon dioxide, indicating its utilization by the microalgae and the occurrence of the purification process. Many literatures discussed the rate and capability of blue-green microalgae to fix CO_2_ via the photosynthesis process. The BG11-N media exhibited a significant (p ≤ 0.05) high ratio of outlet H_2_/CO_2_ when compared with other culture media. It may be attributed to the microalgae exploiting CO_2_ in the gas mixture to perform the photosynthesis process to provide energy for the production of nitrogenase enzyme and fix nitrogen to compensate for nitrogen deprivation [67]. On the other hand, dist.H_2_O media exhibited a significant (p ≤ 0.05) reduction in the outlet H_2_/CO_2_ may be due to the mineral deficiency and the formation of H_2_CO_3_ (CO_2_ + H_2_O) led to of that reduced the pH level and retarded the microalgal growth and metabolism [68] mentioned that the optimum pH for the growth of many microalgal species ranged from 7.2 to 8.0. These results were confirmed by determining the inlet and outlet concentration of CO_2_ (Fig. 5a). The amount of outlet CO_2_ was significantly (p ≤ 0.05) less than the inlet one, which means that the microalgal cells consumed an adequate amount of CO_2_ during the upgrading process, which suggested that the gas mixture had been purified and the quality of biohydrogen has been improved. According to [69], *Oscillatoria* sp. and other microalgae exhibited high potential of CO_2_ fixation, and have been broadly used for biogas upgrading and biogas slurry nutrient reduction. The appropriate CO_2_ level depends on the species of microalgae, the layout of the system, and the operational circumstances [70].

On the other hand, the result in (Fig. 5b) showed a decrease in the amount of outlet H_2_ after upgrading in BG11-N^+g^ and dist. H_2_O^+g^ may be due to the activity of the uptake hydrogenase enzyme or the consumption of H_2_ as an electron donor. The uptake H_2_ase (Hup) found in cyanobacteria absorbs H_2_, and interfered with nitrogenase-based H_2_ production [71]. It was mentioned by [72] that green algae (under anaerobic conditions) can either use H_2_ as an electron donor in the CO_2_-fixation process or evolve H_2_ in both the dark and the light. The losing hydrogen in the case of BG11-N medium may be due to inhibition of photosystem II that is under nitrogen deprivation conditions which increases the anaerobic conditions that activate the hydrogenase enzyme. The latter may activate in the reverse direction and increase the uptake of hydrogen. under hydrogen photoproduction conditions (N-poor medium without fixed nitrogen, anaerobic conditions) the degradation of photosystem II proteins of *Lyngbya* sp. was observed [73]. However, in the case of dist.H_2_O as a culture medium, the losing hydrogen may be due to the absence of nutrients and the use of hydrogen by *Oscillatoria* sp. as an energy source. The main physiological function of the uptake hydrogenase is to reutilize and regain the H_2_/electrons. These results came in context with the recent study of [74] that reported the role of *Chlorella vulgaris* in upgrading methane via decarbonation and desulfurization. Many literatures have been discussed the role of microalgae in purification of biogas from impurities as summarized in Table 4, however, to our knowledge, the highest upgrading percentage is mentioned in the current study (95–98% removal of CO_2_).

**Table 4:** The CO_2_ sequestration and upgrading efficiency % from biofuels by different microalgae-based technologies

Biofuel type	Microalgal species	Culture medium	Removal efficiency (%)	.Reference
		BG11	98	
	*Oscillatoria* sp.	BG11-N	99	This study
Biohydrogen		Dist.H_2_O	95	
Biogas	*Chlorella* sp.	Modified Chu13	89.3	[88]
Biogas	*Chlorella vulgaris*	BG11	54.79–74.65	[89]
Biogas	*Chlorella* sp.	BG11	36.74–57.33	[90]
Biogas	*Scenedesmus obliquus*	BG11	49.95–62.31	[91]
Biogas	*Neochloris oleoabundans*	BG11	40.25–54.39	[91]

Cyanobacteria are a multipurpose feedstock with several biotechnological and biorefining potential [45]. In the current study, the increase in microalgal biomass after the upgrading process may pave the way to apply the current microalgal isolate as a feedstock for biotechnological application [70]. The resulting microalgal biomass can be further utilized to produce biofuels or other value-added products via biorefinery strategies. The biomass production was significantly higher (p ≤ 0.05) in the BG11 medium when compared with other media (Table 1). These results agreed with [83], *Chlorella* 359 (a mutant strain) achieved maximum biomass (1.99 g/L) when it was cultivated in a media supplemented with 5% CO_2_. Similarly, the cultivation of *Synechocystis* in 0.6 g L^−1^ of sodium bicarbonate produced 2.24 g L^−1^ of biomass with 0.22 g L^−1^ day- 1 of CO_2_ fixation rate [84]. Cultivation of *Dunaliella salina* in 5 g L^−1^ NaHCO_3_ produced 3.17 g L^−1^ of biomass [85].

Three primary photosynthetic pigments that stand out in cyanobacteria Chlorophylls, carotenoids, and phycobiliproteins, play a critical role in cyanobacterial cell protection. Due to their bioactive properties, such as antioxidant, antitumoral, antiviral, etc., which can be used in the pharmaceutical, feed, and cosmetic industries as well as functional ingredients in food, these pigments have significant biotechnological significance [46]. In addition, their naturally bright colors are quite appealing to the food colorant and textile industries [86]. In the current study, the BG11 medium of *Oscillatoria* sp stimulated high induction of chlorophyll-a and carotenoid pigment. These results come in context with [45]. They documented that microalgal *Plectonema terebrans* BERC10 exhibited high production of chl-a and carotenoid after 15 days of incubation in BG11 medium.

Lipids extracted from algae are a highly valued metabolite due to their unique ability to produce biodiesel [87]. The microalgae *Oscillatoria* sp isolated in the current study could be used as feedstock for biodiesel production and as third-generation feedstock. it was reported that the biodiesel production is nutrient dependent, a high nutrients enhanced the lipid content of *P. terebrans* BERC10 from 33 to 41% [45].

## Conclusion

It could be concluded that, the pure bacterial strains *Bacillus coagulans* (AH1) could produce biohydrogen by using *Calotropis procera* as a substrate, and their production was influenced by the three variables (concentration of *C. procera* as a substrate, pH values, and temperature levels). The hydrogen yield was 3.23mmol H_2_/g dry biomass at pH 9.0 with a substrate concentration of 5% at temperature of 35 °C, which is relatively higher than other previously mentioned pure cultures. A biological upgrade of biohydrogen was achieved using *Oscillatoria* sp. The CO_2_ removal efficiency percentage was recorded 98, 99 and 95% for the three algal cultures media BG11, BG11-N and dist.H_2_O, respectively. The remaining microalgal biomass exhibited pigmentation production capability and biomass feedstock for promising biodiesel production.

## Data Availability

The authors confirm that the data of the current study are available from the corresponding author upon reasonable request.
